# Tpgen: a language model for stable protein design with a specific topology structure

**DOI:** 10.1186/s12859-024-05637-5

**Published:** 2024-01-23

**Authors:** Xiaoping Min, Chongzhou Yang, Jun Xie, Yang Huang, Nan Liu, Xiaocheng Jin, Tianshu Wang, Zhibo Kong, Xiaoli Lu, Shengxiang Ge, Jun Zhang, Ningshao Xia

**Affiliations:** 1 School of Informatics, Institute of Artificial Intelligence, Xiamen University, No. 422 Siming South Rd, Xiamen, 361005 China; 2 National Institute of Diagnostics and Vaccine Development in Infectious Diseases, State Key Laboratory of Molecular Vaccinology and Molecular Diagnostics, Collaborative Innovation Centers of Biologic Products, Xiamen University, No. 422 Siming South Rd, Xiamen, 361005 China; 3 School of Public Health, Xiamen University, No. 422 Siming South Rd, Xiamen, 361005 China; 4 School of Life Sciences, Xiamen University, No. 422 Siming South Rd, Xiamen, 361005 China; 5 Information and Networking Center, Xiamen University, No. 422 Siming South Rd, Xiamen, 361005 China; 6 State Key Laboratory of Vaccines for Infectious Diseases, Xiang An Biomedicine Laboratory, No. 422 Siming South Rd, Xiamen, 361005 China

**Keywords:** De novo protein design, Protein topologies, Transformer, LSTM, Neural network

## Abstract

**Background:**

Natural proteins occupy a small portion of the protein sequence space, whereas artificial proteins can explore a wider range of possibilities within the sequence space. However, specific requirements may not be met when generating sequences blindly. Research indicates that small proteins have notable advantages, including high stability, accurate resolution prediction, and facile specificity modification.

**Results:**

This study involves the construction of a neural network model named TopoProGenerator(TPGen) using a transformer decoder. The model is trained with sequences consisting of a maximum of 65 amino acids. The training process of TopoProGenerator incorporates reinforcement learning and adversarial learning, for fine-tuning. Additionally, it encompasses a stability predictive model trained with a dataset comprising over 200,000 sequences. The results demonstrate that TopoProGenerator is capable of designing stable small protein sequences with specified topology structures.

**Conclusion:**

TPGen has the ability to generate protein sequences that fold into the specified topology, and the pretraining and fine-tuning methods proposed in this study can serve as a framework for designing various types of proteins.

**Supplementary Information:**

The online version contains supplementary material available at 10.1186/s12859-024-05637-5.

## Introduction

The function of a protein is closely related to its structure. Designing a protein sequence that is capable of folding into a specific structure is a crucial step in creating a protein with a defined function. Two main types of protein sequence design methods exist: the first type of method is fixed backbone protein design, where users typically provide a PDB file or other structural information, and the method generates a sequence capable of folding into the provided structure [1–3]. Examples of this approach are the GVP designed by Bowen Jing [4] and the ESM-IF1 method designed by Chloe Hsu named GVPTransformer [5]. These methods model and extract features from protein atomic coordinates, enabling the generation of sequences that can fold into structures using these features. The ProteinMPNN model, developed by D. Baker, has broad applicability in both single-chain and multichain protein designs [6].The objective of this type of design method is to explore a sequence space that can lead to a specific backbone during the folding process. The second type of design method is sequence design based on sequence features or labels [7], where users either provide limited restrictive information or no input at all, and the model autonomously generates valid sequences. An example of this approach is trDesign, which incorporates trRosetta, a tool for predicting protein sequences, into the generation model. It obtains valid sequences through multiple iterations by progressively escaping the noise distribution using the generated sequence [8]. The advantage of de novo protein design lies in the model’s ability to explore a larger sequence space with fewer restrictions.

Deep learning generative models have gained widespread and effective usage in protein design in recent years [9–14]. Indeed, there exists a significant similarity between protein sequence generation tasks and natural language generation tasks [15]. Similar to natural language processing models that learn patterns between text sequences from extensive data and generate new text sequences based on the learned patterns, the recent release of ChatGPT by OpenAI has demonstrated the high potential of language models for the advancement of diverse domains. Likewise, deep learning generative models for protein design must learn patterns within vast amounts of data comprising amino acid sequences and generate novel protein sequences guided by the learned distribution patterns. Consequently, it is reasonable to anticipate that exceptional models in natural language processing, particularly generative models, will also excel in protein sequence design tasks. Notably, autoregressive models that exhibit proficiency in ChatGPT have also been employed for sequence generation [16, 17]. Autoregressive models have the ability to predict the subsequent token based on a given token until the entire sequence is generated. Autoregressive generative models based on a transformer decoder have achieved noteworthy outcomes. ProGen [18], RITA [19], and ProtGPT2 [20] are expansive generative models trained with extensive datasets using the transformer decoder. Progen employs control labels within a sequence to guide the model in generating sequences with specific label attributes, such as protein family, biological process, and molecule function. RITA and ProtGPT2 learn the sequence distribution from a large dataset, enabling the generation of a broader range of valid sequences.

These models often acquire generalized distributions, while exceling in exploring sequence diversity, they face challenges in targeting specific proteins even after fine-tuning on small datasets for downstream tasks, their performance in terms of protein design may not surpass models specifically trained for such tasks, particularly in cases where there is a scarcity of relevant sequences for the target sequence in the natural protein datasets used for training. We will prove this point in our research. Additionally, protein sequence datasets such as UniProtKB and NCBI lack substantial structural information within the keywords or tags accompanying protein sequences. Consequently, models trained with these datasets face challenges in terms of generating protein sequences with desired structures.

Small proteins consist of relatively short amino acid sequences, typically fewer than 100 amino acids, and exhibit remarkable stability. Owing to their concise amino acid sequences, small proteins frequently adopt stable folding states and exhibit robust resistance to diverse environmental conditions [21]. It is easy to obtain high-resolution prediction results for small proteins through computational methods and experimental techniques owing to their short sequences and relatively uncomplicated structural features [22, 23]. Furthermore, small protein designs have the the advantage of being verifiable through high-throughput experiments [24]. The synthesis and expression of small proteins are relatively straightforward, facilitating the feasibility of high-throughput experimental verification. Through the synthesis and screening of numerous designed small proteins, we can swiftly assess the efficacy of our design strategies and iteratively optimize them based on data obtained from bulk experiments. The introduction of multiple chemical functional groups into small proteins is a relatively straightforward process, enabling their potential utilization in therapeutic and diagnostic applications [25]. LongXing Cao successfully designed a small protein capable of binding to specific targets using his self-developed RifDock method. His research demonstrated the ability to encode different binding specificities within simple helices [26].These findings suggest that small proteins possess significant research value and feasibility. RifDock offers a structural dataset comprising over 60,000 small proteins, enabling us to pursue sequence design for small protein structures.

We developed TopoProGenerator(TPGen), a protein sequence generation framework that utilizes a transformer decoder architecture and integrates reinforcement learning and adversarial learning techniques. TopoProGenerator can generate small protein sequences up to 80 amino acids in length that possess the ability to fold into a specified topological structure.TopoProGenerator is an autoregressive model capable of generating the subsequent token by considering the current token (comprising a topological structure label and amino acids) until either the termination symbol is generated or the specified length is reached.

**Fig. 1:**
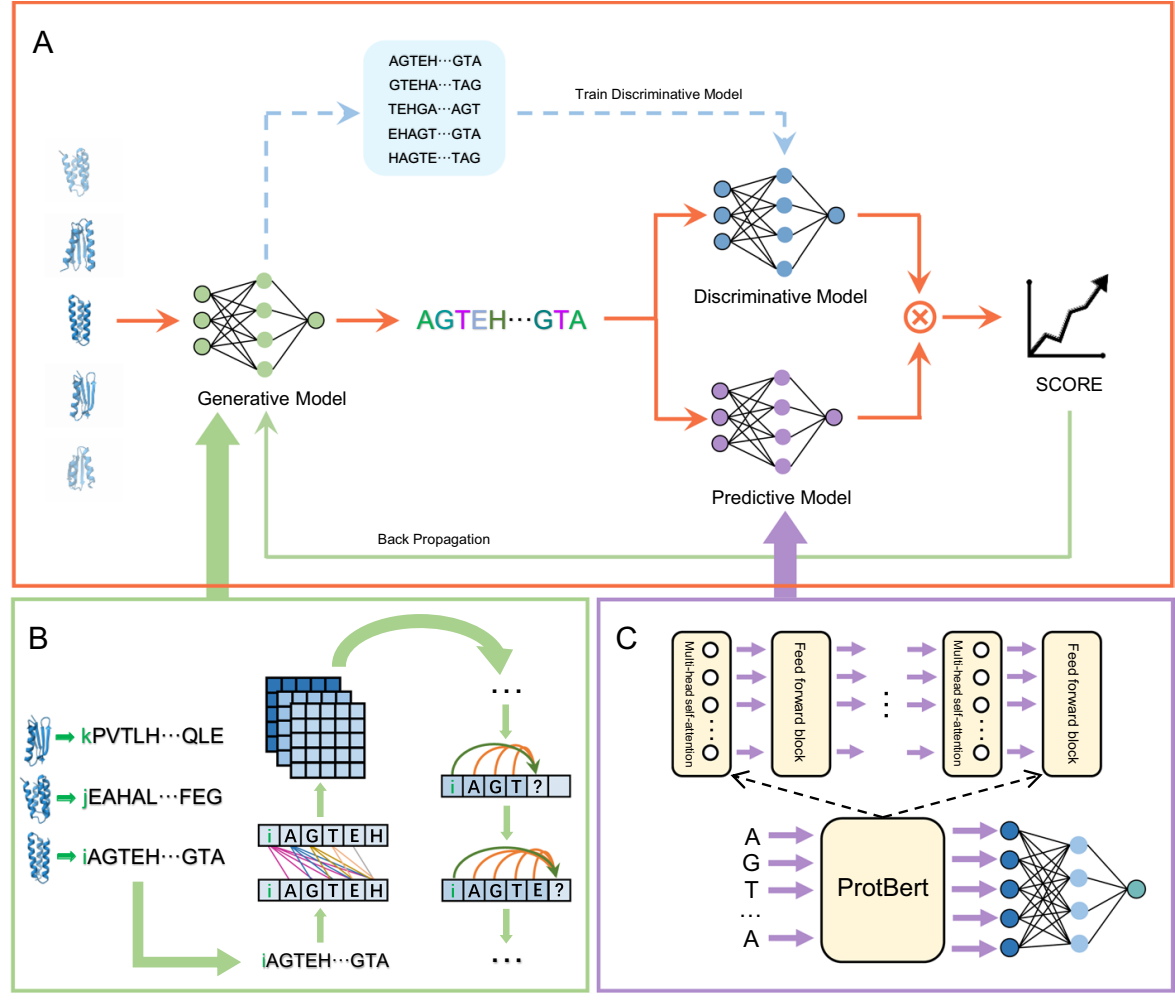
The comprehensive framework for training TopoProGenerator.**A** Framework for model fine-tuning: The first step involves an generative model producing fake sequences with specified topology to train a discriminative model that distinguishes between real and fake sequences. The second step entails the generative model generating sequences, and the discriminative and predictive models providing scores, which are aggregated into a reward for the sequence. Finally, in the third step, the model backpropagates using the reward to optimize the parameters of the generative model.**B** Pretraining process for the generative model: For each sequence, the model constructs features by considering pairwise residue relationships. It utilizes the features of previously generated residues to determine the subsequent residue, maximizing the probability of generating a complete sequence. The first token of the sequence indicates its topology. **C** The predictive model comprises ProtBert and a multilayer perceptron. The sequence input is passed through ProtBert to extract features, which are subsequently fed into the multilayer perceptron to generate stability scores

Initially, TopoProGenerator undergoes unsupervised pretraining with a larger sequence dataset encompassing diverse topological structures. Through iterative optimization, TopoProGenerator learns to predict the probability of the next amino acid based on the original sequence, thereby generating a distribution pattern. Upon pretraining completion, the model is capable of generating a complete sequence by inputting the corresponding topological structure label. To enhance the stability of the generated sequence, we conducted fine-tuning [27, 28] of the model using a relevant smaller dataset (e.g., specifically selecting the HHH portion from the dataset). During the fine-tuning process, we employed reinforcement and adversarial learning techniques. Through reinforcement learning [29, 30], we iteratively trained the generated sequence to enhance its score when using the discriminative model, thereby positively impacting the physical or biological characteristics of the sequence, and we incorporate a pretrained stability prediction model, trained on a large protein dataset with abundant stability data, into the Reinforcement learning framework to enhance the stability of the generated sequences. Similarly, using an adversarial learning approach [31–33], we conducted iterative training of both the generative model and the discriminative model to enhance their performance and maintain adherence of the generated sequences to the amino acid distribution within the dataset.

The primary objectives of this study were to develop and validate TopoProGenerator (TPGen), a novel neural network model, for the de novo design of small, stable proteins with specified topologies. Furthermore, the study aimed to explore the efficacy of integrating a stability predictive model and to assess the impact of various training and fine-tuning strategies on the modelâ€™s generative capabilities.

## Results

The aim of this study was to generate stable small proteins with specified topological structures, necessitating the evaluation of two key aspects. For the first aspect, an assessment of whether the generated small proteins can fold into specified topological structures must be made. For the second aspect, the stability of these proteins must be evaluated. To evaluate and demonstrate the recognized advantages of our design method, we employed methods that were not utilized during the model training. For the first aspect, we utilized a self-written script and employed DSSP for assessment. However, for the second aspect, since there was no general calculation and validation method available, we were unable to rely on the sequence stability prediction model used during the model training. The Rosetta energy scoring function was employed to conduct bulk stability score calculations, yielding statistically significant evaluation metrics. Additionally, molecular dynamics simulations were conducted for the small-scale verification of the optimal outcomes of the model design, as determined by the Rosetta energy score. TopoProGenerator has demonstrated outstanding performance in generating small proteins with diverse topological structures, as the generated proteins adhere more closely to our specified topological structure and exhibit enhanced stability. The model metrics are better than those achieved by the LSTM trained and fine-tuned with the same dataset, and they are significantly superior to the metrics obtained by fine-tuned RITA and random baselines.

### Sequence generation

**Fig. 2:**
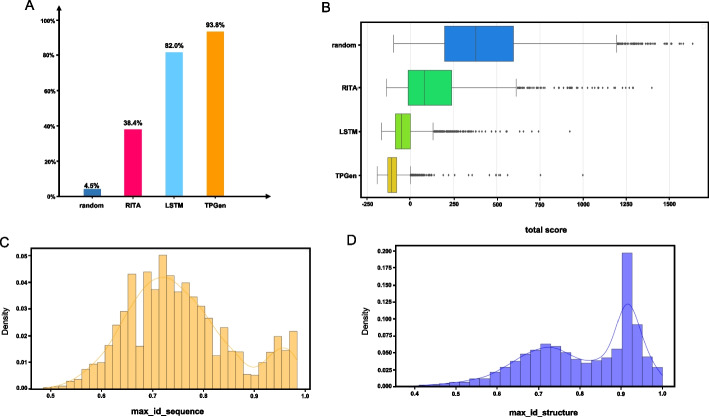
Evaluation results for HHH generation. **A** The proportion of HHH structures in the generated sequences of each model (5000 sequences per model) was assessed using DSSP. TopoProGenerator generated the highest proportion of generated HHH structures,, significantly higher than that for the random baseline and RITA.. **B** The HHH structures (predicted by Omegafold) generated by each model were evaluated using the Rosetta energy scoring function. TopoProGenerator achieved the lowest score, indicating the highest level of stability. The X-axis represents the total energy score. **C** and **D** By utilizing Blast+, we compared each generated sequence with all three-helix datasets for identity matching, retaining the maximum identity value for each sequence (max_id_sequence). By employing the TM-score, we compared each predicted structure of the generated sequences with all three-helix datasets for identity matching, retaining the maximum identity value for each generated structure (max_id_structure)

To evaluate the models’ capability to generate a specific topology sequence, we selected the HHH sequence, which exhibits the highest frequency in our dataset, as the primary target. We assessed both the TopoProGenerator and LSTM models, which were trained using an identical method, and employed RITA and a random baseline as control models. We fine-tuned both the transformer-based TopoProGenerator and LSTM models using our method with the selected HHH dataset. The fine-tuned models exhibited enhanced performance in terms of generating the specified topology sequence and stability-related indicators compared to those of the original models (Table 1). Additionally, we fine-tuned RITA using Nathansen’s fine-tuning pipeline [34] with the same HHH dataset, stopping the training upon convergence. Subsequently, each of these models generated 5,000 small protein sequences, each with a length of 60 amino acids (Additional file 1: Figure S3). Both the TopoProGenerator and LSTM models utilized HHH as the control tag. The topology structures of the generated results were evaluated using DSSP, revealing that the TopoProGenerator and LSTM models outperformed the random baseline and RITA in terms of HHH sequence generation (Fig. 2A).

### Verification of topology structure

**Fig. 3:**
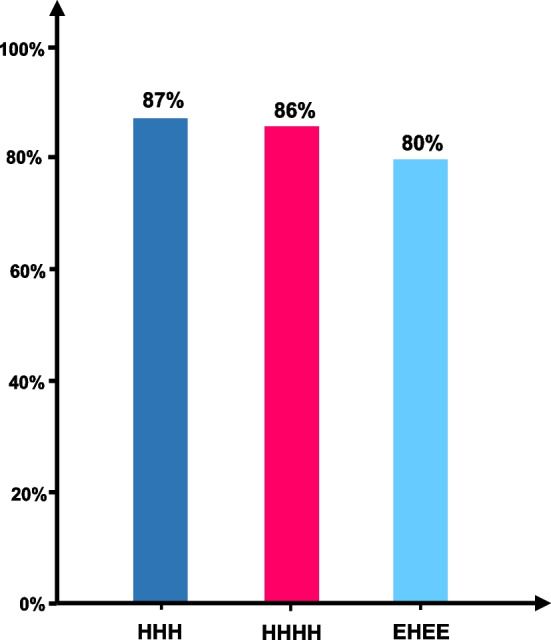
In the designed protein sequence, the proportion of topological structures that conform to the specified topological structure

We used AlphaFold2 to evaluate the sequences we generated. On the one hand, AlphaFold2 has accuracy comparable to that of experimental methods such as cryo-EM(CASP15:92.4%, especially for small protein sequences). On the other hand, AlphaFold2 is relatively fast and can provide meaningful evaluation results. We selected 100 sequences each with HHH, EHEE, and HHHH topology from the design results, predicted the structure with AlphaFold2, and selected the results with the highest plddt. We then computed the topology structure through DSSP. The results showed that the structures predicted from the sequences generated by TPGen largely conform to our specified topology structure labels (Fig. 3).

### Stability evaluation

For the stability evaluation, we select 3000 results with HHH topology structure from the generated sequences. Each of these topologies consisted of 60 amino acids. Initially, we employed Rosetta to relax the selected structures, thereby eliminating unstable conformations, followed by scoring using the Rosetta energy function. Existing protein stability evaluation tools typically compute free energy for mutated sequences, rendering them unsuitable for the artificially composed sequences with highly diverse amino acid compositions in this article. While the Rosetta energy function score may not be a stringent stability evaluation method, it can serve as a stability index for proteins with the same sequence length, allowing us to acquire a substantial number of sequence stability scores to establish statistically significant differences [20]. The Rosetta energy scoring results reveal that the protein sequences generated by TopoProGenerator exhibit superior stability and surpass the results generated by both RITA and the random baseline by a considerable margin(Fig. 2B).

**Fig. 4:**
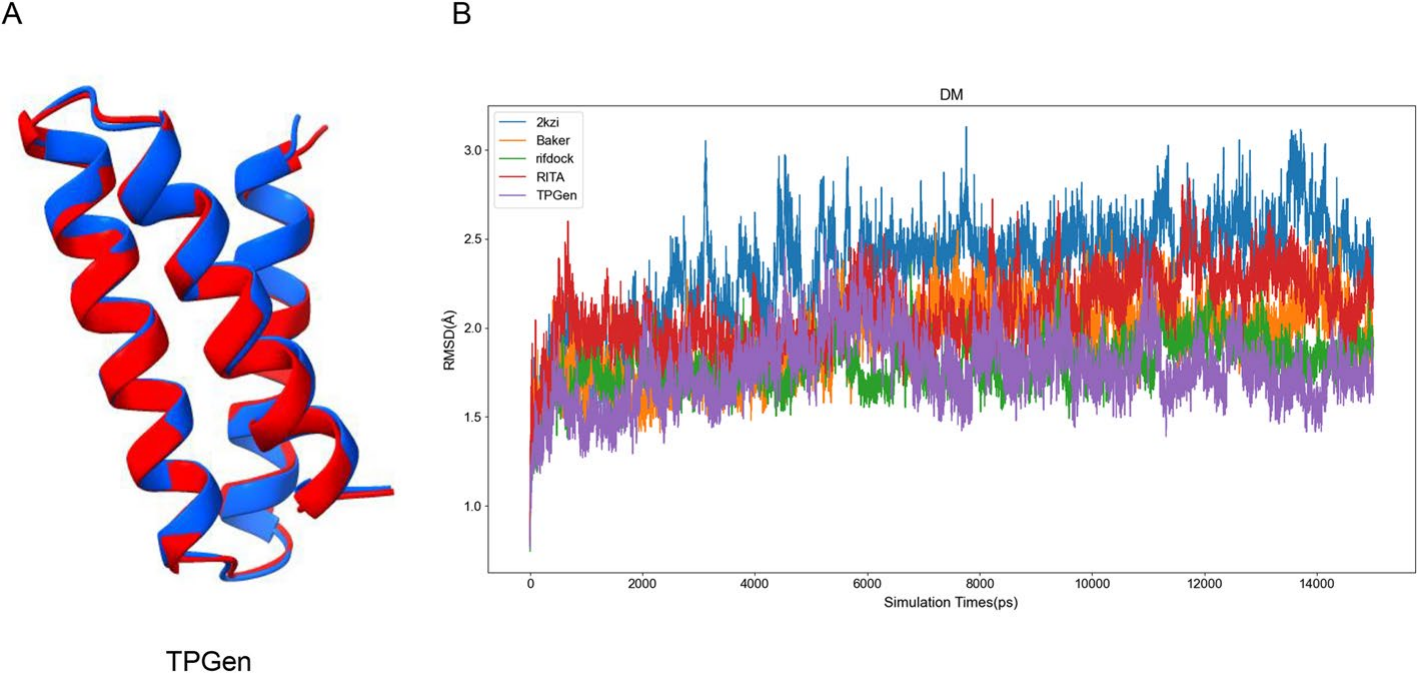
Evaluation of the results for the stability of the sequences using molecular dynamics simulations. **A** Comparing the last frame of molecular dynamics simulation with the original structure, the generated results of TopoProGenerator showed a high degree of identity. **B** From Baker’s designed small proteins, our designed small proteins, RITA’s designed small proteins, and the RifDock HHH dataset, we selected 10 sequences with the lowest Rosetta energy scores. And we selected natural HHH protein 2kzi as the natural baseline. Subsequently, we performed molecular dynamics simulations to compare the selected sequences based on the analysis of the RMSD curve and determined the most favourable sequence. The unit of time is nanoseconds(ns), and the graph is plotted by averaging the values of all data points within each nanosecond(ns)

In our work, molecular dynamics simulation was employed to assess the structural stability of the designed proteins [35, 36]. Based on the Rosetta energy scores, we chose the 10 sequences with the lowest scores from the TopoProGenerator and RITA-designed results, along with the RifDock dataset utilized for training. Simultaneously, we selected the 10 most stable sequences from the small proteins designed by D. Baker [37]. In addition, we selected natural HHH protein 2kzi as the natural baseline. We used AlphaFold2 to predict the structures of the selected sequences and performed molecular dynamics simulations. We evaluated the root-mean-square deviation (RMSD) of the protein conformation at the current moment in comparison to the initial conformation during the molecular dynamics simulation. A stable and consistently low RMSD curve indicates minimal changes in a protein structure throughout the simulation, signifying its relative stability [36]. Each model preserves its best result for comparative analysis with other model (Fig. 4).Regarding stability, the sequences generated by TopoProGenerator exhibited superior performance compared to that of the protein sequences designed by RITA and D. Baker, and it is better than natural protein 2kzi. However, TopoProGenerator exhibited slightly inferior performance when considering the most stable results in the RifDock dataset.

These findings indicate that TopoProGenerator has learned the connections between residues and topological structures at the sequence level, as well as the low-energy relationships inherent in valid protein sequences.

### Diversity evaluation

**Fig. 5:**
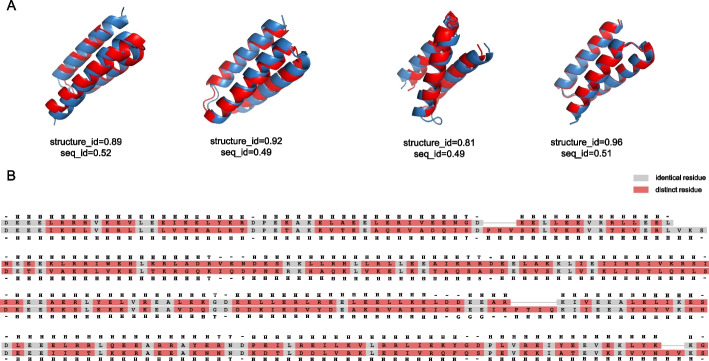
**A** Despite significant sequence differences, the generated proteins exhibit remarkable structural consistency compared to that of the proteins in the dataset, which is a noteworthy result. It is important to highlight that the training process relies solely on topological structure labels, as no other structural information is provided. Structure ID and seq ID correspond to the maximum structural consistency and sequence consistency in the dataset, respectively. **B** Sequence alignment of sequences in (A). The above is the sequence from the RifDock backbone library, and the following is the sequence we designed. *The identical residues are denoted in grey, while the distinct residues are highlighted in red. Concurrently, we have annotated the secondary structure type corresponding to each residue, as deduced from DSSP, where G signifies a* $3_{10}$ -*helix, H represents an* $\alpha$ -*helix, and T indicates a hydrogen*-*bonded turn*

We assessed the diversity of generated sequences using Blast+ [38] and MMseqs2 [39].The HHH sequences generated by TopoProGenerator exhibited significant dissimilarity from the sequences in the dataset(Fig. 2C). Additionally, we evaluated the structural diversity of the generated sequences within the same topology by employing the TM-score to compare the maximum identity of the generated structures with those in the database. The structure generated by TopoProGenerator significantly differs from the structures in the database despite sharing the same topology. Based on the TM-score definition, a TM-score exceeding 0.5 indicates that the structures have an identical topology. Our design results exhibited TM-scores predominantly ranging from 0.5 to 0.9, signifying that the proteins generated by TopoProGenerator share the same topologies as those in the database while possessing noteworthy distinctions (Fig. 2D). Additionally, MMseqs2 was employed to cluster the HHH training dataset and the generated HHH sequences individually. The generated sequences demonstrated greater diversity compared to that of sequences in the training dataset (Additional file 1: Table S1). Furthermore, it was discovered that certain sequences generated by TopoProGenerator showed low similarity with sequences in the training set at the sequence level but high similarity at the structural level (Fig. 5). This finding suggests that TopoProGenerator explores the potential of diverse sequences within the same structural framework. In other words, our model traverses a broader sequence and structure space while maintaining the same topological structure.

### Other topological structures

To assess the model generalizability across the entire dataset, we employed TopoProGenerator to generate additional topological structures for evaluation purposes. For verification, we chose HHHH, the second-most abundant topological structure in the dataset, and EHEE, a less frequently occurring topological structure. We utilized both TopoProGenerator and LSTM models for generation and evaluated them using the same methodology as that employed for HHH. By utilizing the corresponding topological structure tags as control labels, TopoProGenerator generated sequences that exhibited a proportion of corresponding topological structures exceeding 85%. Notably, during the generation of EEHE, TopoProGenerator produced a significantly higher proportion of EHEE sequences compared to LSTM. This observation implies that the Transformer Decoder exhibits superior capability in capturing the mapping relationship between topological structure tags and amino acids, particularly when operating with limited data sets. Based on the Rosetta energy function score, the TopoProGeneratorâ€™s design results surpass the random baseline, providing evidence of its adherence to the distribution pattern of proteins exhibiting low energy levels. TopoProGenerator has an excellent generation ability on the topological structure outside of HHH (Additional file 1: Figure S1). We conducted the same molecular dynamics evaluation process as HHH for EHEE and HHHH (Additional file 1: Figure S5),The results show that the stability of HHHH generated by TPGen is superior to that of the bacbackbone library. We believe this is due to the fact that the data set of HHHH is richer in data volume compared to EHEE, and the model has been trained more thoroughly.

### Ablation experiment: Fine-tuning of different parameters

We conducted experiments to assess the performance of the fine-tuned models with and without the sequence stability predictive model. Various methods were employed to process the stability predictive model scores. The stability predictive model scores were processed using Equation (2), and the values of $\lambda 1$ and $\lambda 2$ were adjusted for different experiments (Table 1).

**Table 1:** Evaluation of various treatment approaches and their respective scores for fine-tuning sequence stability prediction models

	Pretrain ^a^	$1.2-0.6$^b^	$1.0-0.0$^c^	OriScale ^d^	NoPredictor^e^
Proportion of HHH	91.35%	93.82%	90.82%	91.42%	91.54%
Proportion of stable proteins ^f^	63.63%	69.90%	64.35%	62.41%	62.51%
Average stability score of HHH	1.05	1.12	1.06	1.04	1.04

^a^ Model without fine-tuning

^b^ Fine-tuning with $\lambda 1$=1.2 and $\lambda 2$=0.6

^c^ Fine-tuning with $\lambda 1$=1.0 and $\lambda 2$=0.0

^e^ Fine-tuning achieved by directly multiplying the stability scores with the discriminative model scores

^d^Fine-tuning with only the discriminative model

^f^ Proportion of stable proteins in HHH

**Fig. 6:**
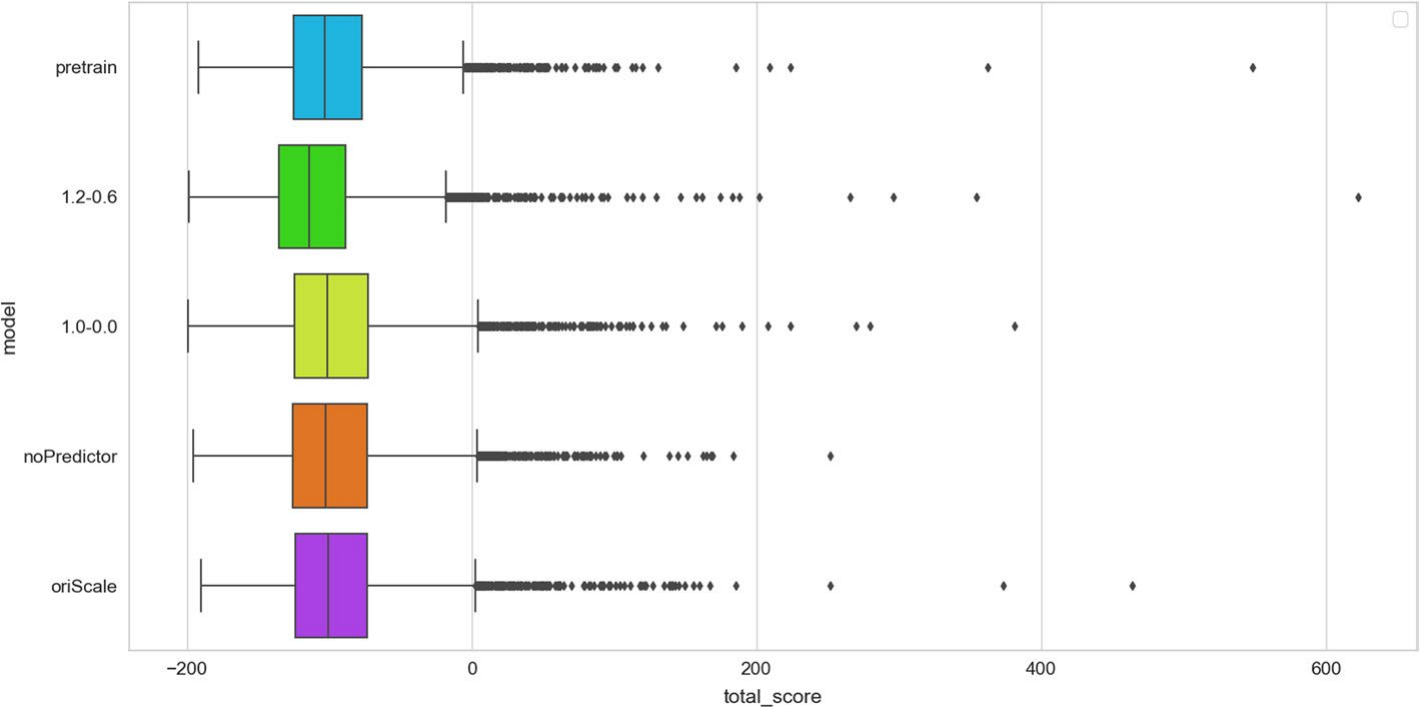
Evaluation results of ablation experiments for various treatments of sequence stability predictor scores are presented. We conducted a unified evaluation process for different treatments based on the predicted stability scores of the sequences, using the Rosetta score function to obtain the results. The X-axis represents the Rosetta score function scores, where lower scores indicate greater protein stability. The Y-axis represents various treatments

The results indicated that the optimal performance was achieved when $\lambda 1$=1.2 and $\lambda 2$=0.6 (Fig. 6). This can be partly attributed to the fact that the sequences in the HHH dataset represent the most stable portion. Therefore, fine-tuning on this dataset should prioritize the preservation and enhancement of stability in the generated sequences, rather than eliminating unstable outcomes.

## Conclusions

In summary, our study demonstrates that transformer-based conditional language models trained solely on sequence data annotated with topological structure information are capable of generating small protein sequences that can adopt the desired topological structure. These generated sequences exhibit both high stability and diversity, effectively exploring the sequence space within the specified topological structure. Moreover, the comparison with RITA trained with a large protein sequence dataset highlights the efficacy of our study in achieving our objective. It substantiates that training with specific and relatively small datasets can be successful when protein structure data are inadequate or when the goal is to generate proteins with limited representation in the dataset.

We further explored the transformer’s capacity to learn the mapping between a topological structure and sequence. In contrast to prior studies, we employed topological structure identifiers, e.g., we used HHH rather than a single lowercase letter such as “i” to represent the topological structure of a sequence. Our objective was to investigate whether the model could capture the relationship between a single helix or fold and a sequence capable of folding into a topological structure. The sequences in the dataset were transformed into the format of <xxx>ATG...GTA, where “xxx” denotes the topological structure identifier. Lowercase letters were employed to represent topological structures, allowing for the distinction between identifiers and amino acids. The separation of the topological structure identifier from the amino acid sequence was indicated using $<>$.

The model was pretrained using the same methodology as that for TopoProGenerator. Initially, we tasked the model with generating HHH sequences, and subsequent validation confirmed its ability to generate the existing topological structure present in the dataset. Next, we instructed the model to generate HEHE and HEEEH sequences, which are topological structures that are not present in the dataset. Despite the proteins generated by the model were identified by DSSP as possessing these topological structures, their occurrence rate was relatively low, approximately 3-5$\%$. Concurrently, we investigated the presence of these two topological structures in the sequences previously generated by TopoProGenerator. Our findings revealed an almost negligible occurrence of these topological structures in the sequences generated by TopoProGenerator. This observation suggests that the model has indeed acquired certain associations between a topological structure and sequence; however, the extent of this learning is limited.The structure of HEHE was predicted using Alphafold2, revealing a higher abundance of loops compared to that in typical proteins. We employed molecular dynamics simulations to assess the stability of the designed HEHE results, and only a limited number exhibited a certain level of stability. The overall stability level was notably lower compared to HHH (Additional file 1: Figure S2).

## Methods

TopoProGenerator comprises transformer decoders and undergoes training in two stages: pretraining and fine-tuning. In the pretraining phase, the model is trained with a sequence dataset to regenerate each training datum (Fig. 1B). The fine-tuning process involves utilizing a pretrained predictive model to derive stability scores based on the sequence and a discriminative model to assess the distribution of generated sequences and fine-tuning datasets. The model undergoes fine-tuning by employing reinforcement learning and adversarial learning methods (Fig. 1A).

### Data processing

To train TopoProGenerator, we processed the protein backbone library obtained from RifDock. Since the backbone library of RifDock was generated through Rosetta predictions, which may not be highly accurate, we incorporated Omegafold for verification purposes. Omegafold is a single-sequence structure prediction tool capable of accurately predicting high-resolution protein structures based solely on a single sequence. Notably, it can effectively predict orphan proteins, which do not belong to any protein family, aligning with the specific requirements for predicting artificial proteins. All sequences were extracted from the backbone library, and their structures were subsequently predicted using Omegafold. Next, we compared the predicted structures with the original structures using the TM-score metric and retained sequences with TM-scores exceeding 0.9 as our original dataset. To differentiate the sequences from amino acids, we labelled the sequences based on their topological structure, assigning a single lowercase letter to represent each distinct topological structure. For instance, “i” denotes HHH, “j” represents HHHH, and so forth. The dataset encompassing all topological structures served as the pretraining dataset, while the dataset with a single topological structure was utilized for fine-tuning.

### Stability predictive model

We utilized the dataset provided by Jedediah M. Singer [40], which comprises sequence stability scores obtained through high-throughput assays described by Rocklin [41], to evaluate protein stability. The authors combined multiple protein datasets into a standardized benchmark dataset for protein sequence stability, encompassing a total of 280,000 sequences, suitable for joint training purposes. The model architecture for predicting sequence stability comprises ProtBert and a multilayer perceptron(Fig. 1C). A self-supervised training process was applied to ProtBert, introduced by Ahmed Elnaggar, using a large-scale sequence dataset [42]. We evaluated all sequences in the dataset using the stability predictive model and plotted distribution maps according to different topological structures (Additional file 1: Figure S4).We found that within the Rifdock backbone library, HHHH and HHH exhibit the highest stability, followed by EHEE and HEEHE, and then proteins with other topological structures.

A random sample of 10,000 sequences from the dataset was chosen as the test set. Subsequently, we assessed the model’s performance with the test set after training it to convergence. The model demonstrated remarkable accuracy and stability.

Then, we employed the stability predictive model to improve the fine-tuning process during the reinforcement learning phase, with the aim of optimizing the stability of the generated sequences.

### Conditional language modelling

Consider a as ($a_1$, $a_2$, $a_3$,..., $a_{na}$), which represents an amino acid sequence of length $n_{a-1}$, with a “sequence end” mark (”$\backslash$”) appended to create a sequence of length *na*. *t* denotes the control label for the topological structure, which is added at the beginning of the sequence to guide the model in generating sequences with predefined topological structures. Thus, $x = [t;a]$ represents a sequence obtained by prepending the control label to the amino acid sequence, and this composite sequence is utilized for training purposes. The probability of this composite sequence having a length of $n = n_{a}+1$ is denoted as *P*(*x*). The problem of generating x is decomposed in language modelling, wherein each subsequent token is predicted individually [43]. In this model, only amino acids are predicted. A network with parameters $\theta$ is trained to minimize the negative log-likelihood on the dataset $D={x_1, x_2, x_3,..., x_{|D|}}$.

1 $$\begin{aligned} L(D)=-\frac{1}{|D|}\sum _{K=1}^{|D|}\frac{1}{n^k}\sum _{i=1}^{n^k}logp_{\theta }(x_i^k|x_{<i}^k) \end{aligned}$$

The generation model employs an autoregressive architecture, wherein the subsequent token is generated based on the preceding input. By using the control label *t*, the protein a is generated by sampling amino acids iteratively: $p\theta (a_1|t)$, $p\theta (a_2|a_1,t)$,..., $p\theta (a_i|a_{<i},t)$, until the model determines the position of the “sequence end” marker.

We employed neural network architectures based on transformers to build TopoProGenerator. Transformers utilize multiple stacked layers to capture contextual relationships within sequences, with each layer incorporating a self-attention mechanism. The self-attention mechanism enables the establishment of contextual relationships within a sequence, specifically the connections between amino acids. Previous studies have indicated that as the number of self-attention layers increases, the model progressively learns more intricate internal relationships among a protein [44]. Furthermore, previous models such as MRFs [45] and Potts models [46] have demonstrated a relationship between transformer-based methods and co-evolution methods used in sequence design. TopoProGenerator serves as a transformer decoder employed for autoregressive generation, producing sequences token by token, with each subsequent token conditioned on all previously generated tokens.

Additionally, we employed a neural network architecture based on LSTM for the same training and validation as those of a control model. LSTM is a specialized type of recurrent neural network (RNN) [47] that utilizes a cell state $C_t$ to store and transfer the current LSTM state information to the subsequent LSTM unit at each time step. This architecture resolves the issue of long-term dependencies in RNNs and is effective in terms of generating contextually connected sequences.

### Training

The training process consists of pretraining and fine-tuning stages. The model trains with the complete sequence dataset to generate sequences aligned with specific topological structure labels. To fine-tune with a particular topological structure dataset and enhance our model’s generation performance on that structure, we employed a combination of reinforcement learning and adversarial learning techniques. The training history of all models can be obtained in (Additional file 1: Figure S6-S9) and Checkpoint of TPGen.

### Pretraining

The transformer architecture of TopoProGenerator comprises 5 layers, with each layer consisting of 8 self-attention heads. The dimensions of *q*, *k*, and *v* in the self-attention mechanism are set to 32, and the feedforward layer has a dimension of 512. TopoProGenerator is trained to minimize the negative log-likelihood as defined in Equation 1. All sequences are padded to a length of 80, and the padded tokens are excluded from generating new amino acids. The trained model has the capability to generate protein sequences by providing specific topological structure labels, such as “i” to generate “HHH”. The LSTM architecture consists of 10 layers, which were selected after multiple training sessions, with each layer containing 1024 nodes. The training process follows a similar approach as that of the transformer.

### Fine-tuning

Prior studies have demonstrated that incorporating a discriminative model or scorer to guide the sequence generation model can introduce a bias towards generating higher-scoring outcomes [48]. Our fine-tuning framework utilizes the concept of adversarial learning and comprises a generative model *G* and a discriminative model *D*. Initially, negative samples are generated by *G*, while positive samples are obtained from the selected dataset to train *D* to distinguish between the two types of samples. Subsequently, *G* is trained to generate sequences that serve as input into *D*. The resulting score *D* from *D* is combined with the score of the sequence stabilizer *P* as feedback, guiding *G* in producing sequences that align with the distribution of the selected dataset and exhibit high stability. The reward function is defined as follows:

2 $$\begin{aligned} \begin{aligned}\;&if P(x) >= 1:\\&R(x) = \lambda 1 \times D(x) \\&else: \\&R(x) = \lambda 2 \times D(x)\\ \end{aligned} \end{aligned}$$

where *D*(*x*) represents the score assigned by the discriminative model *D* to the generated sequence *x*, and *P*(*x*) represents the score assigned by the stability predictive model *P* to the generated sequence *x*.The final reward score is *R*(*x*) Sequences *x* with *P*(*x*) greater than or equal to 1.0 are regarded as stable sequences. The reward function is applied differently to stable and unstable sequences, and the objective of fine-tuning training is to maximize the reward function.

To address this issue, when generating sequences with autoregressive models (LSTM and transformer), discrete distribution sampling (argmax) is used to obtain tokens one by one from the model’s output. However, backpropagation cannot compute gradients for sampling. Researchers have employed reinforcement learning [49] or Gumbel-softmax reparameterization [50] to address this issue. Given the similarity between the process of generating sequences with autoregressive models and policy gradient [51] methods in reinforcement learning, the amino acid space can be regarded as the action space, the generated partial sequences can be considered as the environmental state *s*, and the combined score of p and d can be interpreted as the reward *r*. Throughout multiple iterations of reinforcement learning, the generator progressively enhances the score combination *R*. TopoProGenerator employs the policy gradient method of reinforcement learning to compute gradients for adversarial learning and backpropagation. Its reward function combines the discriminative model and the stability predictive model scores, providing guidance to the generator in terms of generating valid and stable sequences. During the fine-tuning process, we choose sequences with predefined topological structures from the training set to create the fine-tuning dataset, which serves as positive samples for the discriminative model. Cosine annealing and warm-up techniques were employed to adjust the learning rate during the fine-tuning process.

The pretrained model was fine-tuned with a sequence dataset that exclusively contains the desired topological structures. The generated sequences were further fine-tuned utilizing a sequence discriminative model and a stability predictive model. The Adam optimizer [52] was employed, and the model was trained for a total of 50 epochs with a learning rate of 0.000001. During each epoch, 150 sequences were trained, and we chose the checkpoint with the lowest average loss (negative reward value) for sequence generation. For further evaluation, the model was instructed to generate 5000 sequences.

### Random baseline

Protein sequences consisted of 60 amino acids are randomly generated by selecting one amino acid from a pool of 20 amino acids for each residue position. The resulting sequence can be considered completely random.

### Evaluation

#### Evaluation process

We utilized Omegafold to predict the structures of the generated sequences and used DSSP to determine their topological structures and select the specified topological structures. We performed relaxation using Rosetta to eliminate any unreasonable conformations in the structure. Finally, the generation results were evaluated utilizing the Rosetta energy function, sequence stabilizer, Blast+, MMseqs2, and molecular dynamics tools.

#### Selecting topological structures using DSSP

DSSP can deduce the secondary structure at the residue level (e.g., H or E) based on the protein’s structural information. We have developed a proficient script for analyzing the outcomes obtained from DSSP. Through evaluating the patterns of consecutive H or consecutive E, we ascertain the overall topological structure of the protein, which determines its specific topological structure type (e.g., HHH, HHEH, etc.).

#### Scoring with the Rosetta energy function

The Rosetta energy function is derived by assigning weights to a range of measurable geometric statistics and classical physical interactions. It assesses the magnitude of the interaction energy between atoms based on the provided atomic coordinates. The energy function incorporates the up-to-date full-atom model(ref2015) [53, 54] and includes the cart_bonded term weighted at 0.625. Refer to S2 for a detailed explanation of the scoring components used in the energy function.

#### Evaluating sequence diversity using Blast+ and MMseqs2

To assess the diversity of the generated sequences, we employed the multiple sequence alignment tools, Blast+ and MMseqs2. By using Blast+, we were able to conduct pairwise identity comparisons between the generated sequences and the training set, retaining the highest identity value (max_id) for each comparison. By employing MMseqs2, we were able to cluster the generated sequences and utilize the resulting number of clusters as an analysis index.

#### Evaluation of structural diversity using TM-score

We utilized the TM-score to compare the predicted structures of each generated HHH sequence with all the HHH skeletons, and selected the highest TM-score as the indicator of structural similarity.

#### Evaluation using molecular dynamics

MD simulations were performed using the NAMD version 2.13 MD package [55]. The integration timestep of the simulation was set to 2 femtoseconds (fs), and the position coordinates (DCD file) were saved every 4 ps for further analysis. Long-range periodic electrostatic interactions were evaluated using the smooth Particle-Mesh Ewald (PME) [56] method, with a real cut-off radius of 10 Ã…. The lengths of all chemical bonds involving hydrogen bonds were constrained by the SHAKE algorithm [57]. CMD simulations were performed at 310 K and the constant temperature was controlled by Langevin dynamics [58] under a pressure of 1 atm [59] maintained using the Nose-Hoover thermostat. Before each production, the system was energy-minimized by 2000 conjugate gradient steps to reduce steric conflicts between water molecules and the protein. The dynamic results were analysed using the VMD program.

### Supplementary Information

**Additional file 1**. Supplementary results.

## Data Availability

The dataset used to train the sequence stability predictive model is available in the zoom repository,Dataset for predictive model. RifDock backbone library can be found at RifDock library. The processed dataset, along with HHH sequences of various lengths generated by TopoProGenerator (including HHH sequences), as well as HHHH and HHEH sequences generated by TopoProGenerator, is available in the zoom repository, Sequence generated by TPGen. The code of TopoProGenerator is available in the github, Code of TPGen.
