# Supplementary material for: Tpgen: a language model for stable protein design with a specific topology structure
Source: BMC Bioinformatics. 2024 Jan 23;25:35. doi: 10.1186/s12859-024-05637-5 (PMC10804651; doi:10.1186/s12859-024-05637-5)
Supplement: Supplementary file 1 — Additional file 1. Supplementary results. [file 12859_2024_5637_MOESM1_ESM.docx]

Article

TPGen: A language model for stable protein design with a specific topology structure

In the format provided by the

authors and unedited

|  | RifDock | TPGen |
| --- | --- | --- |
| Diversity-0.5 | 1172 | 3253 |
| Diversity-0.8 | 4668 | 4830 |

**Table S1:** TopoProGenerator generates “HHH” proteins that exhibit higher sequence diversity compared to that of the three-helix proteins in the RifDock dataset. The sequence datasets were clustered using MMseqs2, employing a minimum identity threshold of 50%/80% and a coverage threshold of 0.8. The diversity measure is determined by the number of clusters containing three or more members, with a higher value indicating a greater degree of library diversity.

| **Function** | **analy** |
| --- | --- |
| cart_bonded | Cartesian bonded potential |
| dslf_fa13 | Disulfide geometry potential. Supports D- and L-cysteine disulfides, plus homocysteine disulfides or disulfides involving beta-3-cysteine. |
| fa_atr | Lennard-Jones attractive between atoms in different residues. Supports canonical and noncanonical residue types. |
| fa_dun | Internal energy of sidechain rotamers as derived from Dunbrack's statistics (2010 Rotamer Library used in Talaris2013). Supports any residue |
| fa_elec | Coulombic electrostatic potential with a distance-dependent dielectric. Supports canonical and noncanonical residue types. |
| fa_intra_rep | Lennard-Jones repulsive between atoms in the same residue. Supports canonical and noncanonical residue types. |
| fa_intra_sol_xover4 | Intra-residue LK solvation, counted for the atom-pairs beyond torsion-relationship. Supports arbitrary residues types. |
| fa_rep | Lennard-Jones repulsive between atoms in different residues. Supports canonical and noncanonical residue types. |
| fa_sol | Lazaridis-Karplus solvation energy. Supports canonical and noncanonical residue types. |
| hbond_bb_sc | Sidechain-backbone hydrogen bond energy. |
| hbond_lr_bb | Backbone-backbone hbonds distant in primary sequence. |
| hbond_sc | Sidechain-sidechain hydrogen bond energy. |
| hbond_sr_bb | Backbone-backbone hbonds close in primary sequence. All hydrogen bonding terms support canonical and noncanonical types. |
| lk_ball_wtd | weighted sum of lk_ball & lk_ball_iso (w1*lk_ball + w2*lk_ball_iso); w2 is negative so that anisotropic contribution(lk_ball) replaces some portion of isotropic contribution (fa_sol=lk_ball_iso). Supports arbitrary residue types. |
| omega | Omega dihedral in the backbone. A Harmonic constraint on planarity with standard deviation of ~6 deg. Supports alpha-amino acids, beta-amino |
| p_aa_pp | Probability of amino acid at Φ/Ψ. Supports only the 20 canonical alpha-amino acids and their mirror images. |
| rama_prepro | Backbone torsion preference term that takes into account of whether preceding amono acid is Proline or not. Currently supports the 20 canonical alpha-amino acids, their mirror-image D-amino acids, oligoureas, and N-methyl amino acids. Arbitrary new building-blocks can also be supported provided that an N-dimensional mainchain potential can be generated somehow. |
| ref | Reference energy for each amino acid. Balances internal energy of amino acid terms. Plays role in design. Supports only the 20 canonical al |
| yhh_planarity | Special torsion potential to maintain tyrosine hydroxyl groups in the plane of the aromatic ring |

**Table S2:** Explanation of the relevant score terms in the Rosetta energy scoring function.


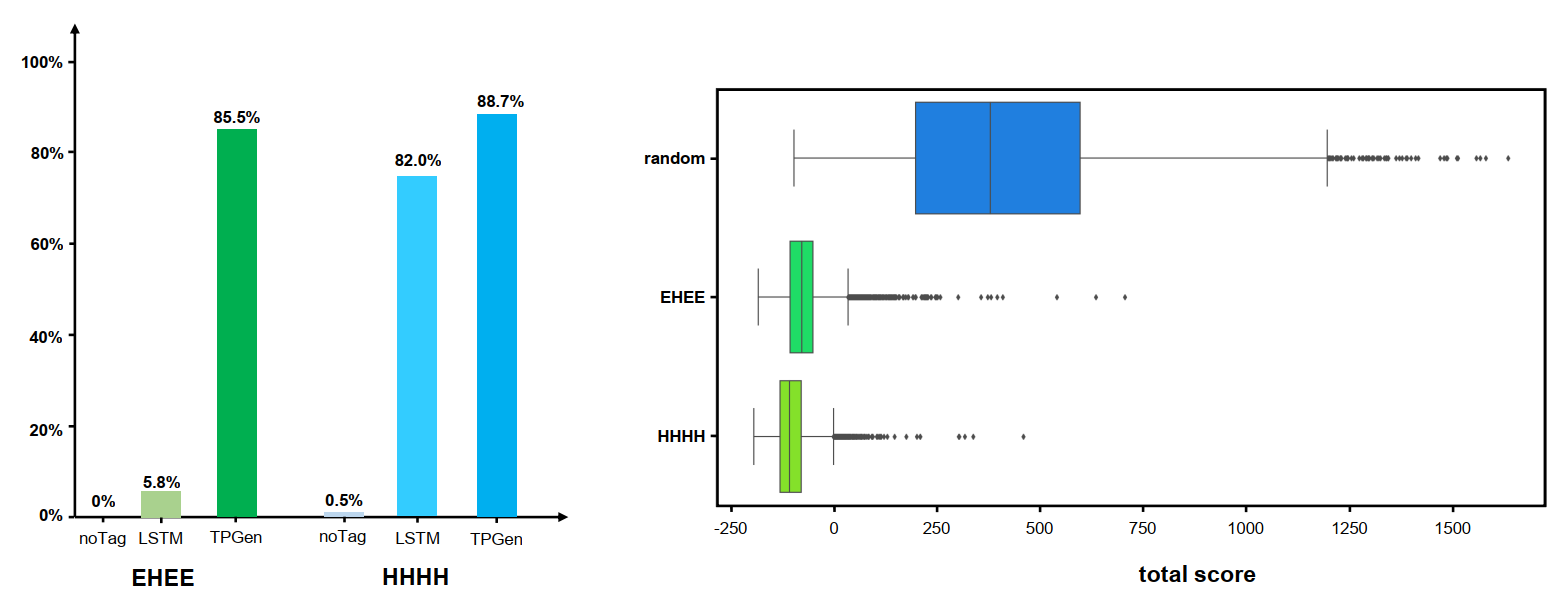


**Figure S1:** Evaluation of the for the models' capability to generate sequences with secondary structures (EHEE/HHHH). We employed three models to generate 5000 sequences labelled with EHEE/HHHH secondary structures as input. The “noTag” model, on the other hand, was trained using a pure sequence set without secondary structure labels. Subsequently, following structure prediction using Omegafold, the results were evaluated as follows: (A) Calculating proportions of the specified secondary structures using DSSP. (B) Scoring the generated secondary structures, specifically EHEE/HHHH, using Rosetta. The obtained scores for both HHHH and EHEE were significantly lower than those of the random baseline, indicating that the models successfully learned the relationships between sequences and their stability.


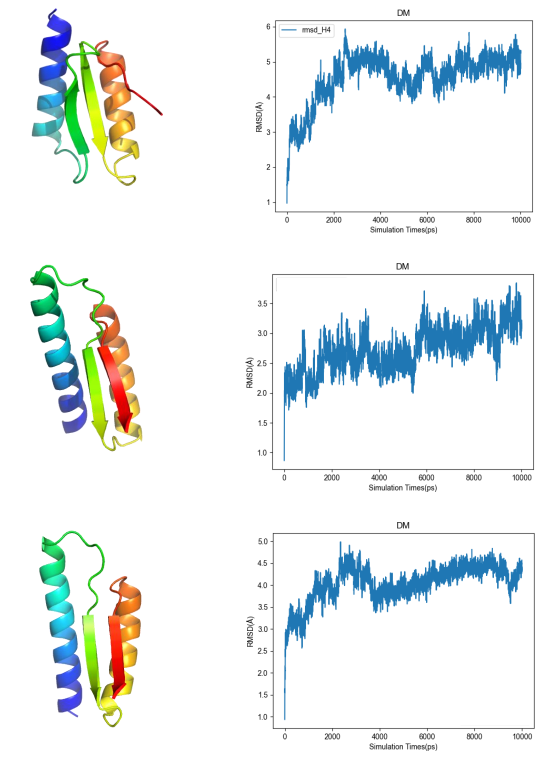


**Figure S2:** HEHE design result. Predict the structure using AlphaFold2 and simulate it using molecular dynamics.

*
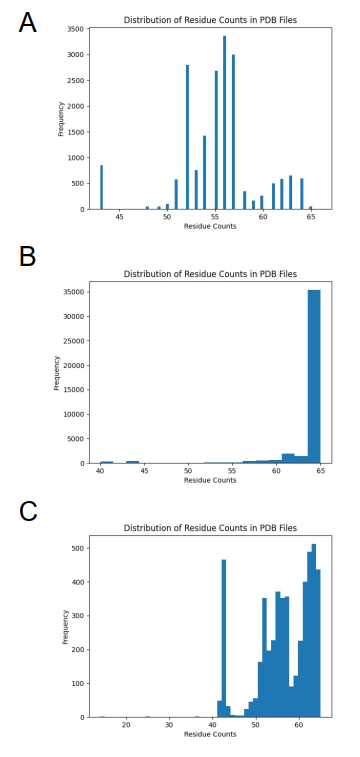
*

**Figure S3**: Length distribution of protein sequences in the dataset and protein sequences generated by TPGen: (A) Length distribution of sequences with a topology of HHH in the dataset. (B) Length distribution of sequences with a topology other than HHH in the dataset. (C) Length distribution of sequences generated by TPGen.

The reason for generate sequence length of 60: (1)In our dataset, the sequence length of HHH is concentrated around 55, while the sequence length of other topological structures is concentrated around 64-65. Since TPGen's pre-training is based on the entire dataset, choosing a length of 60 can take into account both the distribution of HHH and other topological structures. (2)The length distribution in the generated sequences has two centers at 55 and 65. Selecting a length of 60 can take into account the distribution characteristics of the generated sequence length. (3)Sequences that are too short are inadequate to form stable tertiary structures, while sequences that are too long are difficult to be screened through yeast surface display libraries using synthetic gene chips. Therefore, we made a compromise on this length.

*
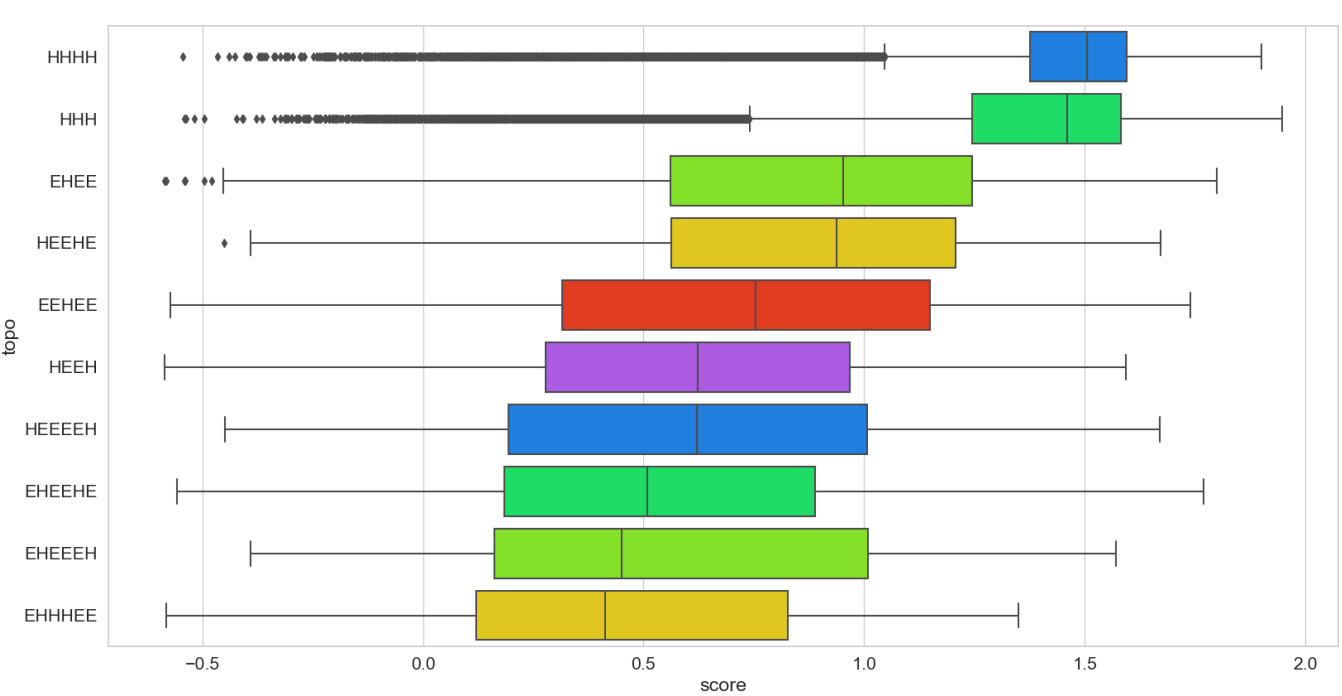
*

**Figure S4**: Stability evaluation of sequences with different topological structures using stability predictive models

*
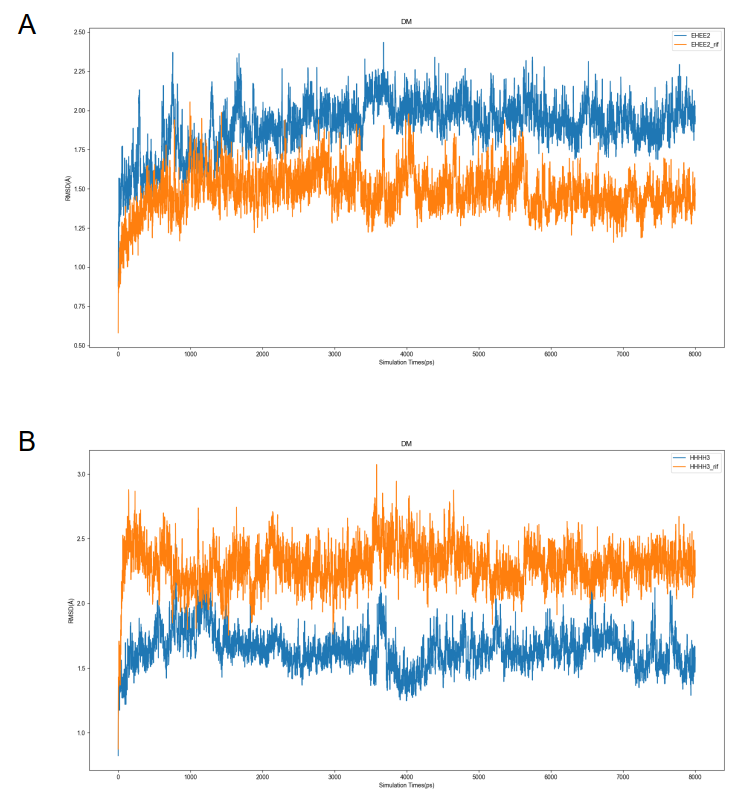
*

**Figure S5**: Simulate design of EHEE and HHHH using molecular dynamics. We used Alphafold2 to predict the structures of the top 10 sequences with the highest Rosetta energy score in the design results and performed molecular dynamics simulations, retaining the best simulation results. We also performed the same process on the top 10 sequences selected from the RifDock backbone library, retaining the best simulation results, and compared the two results. (A)EHEE. (B)HHHH.

*
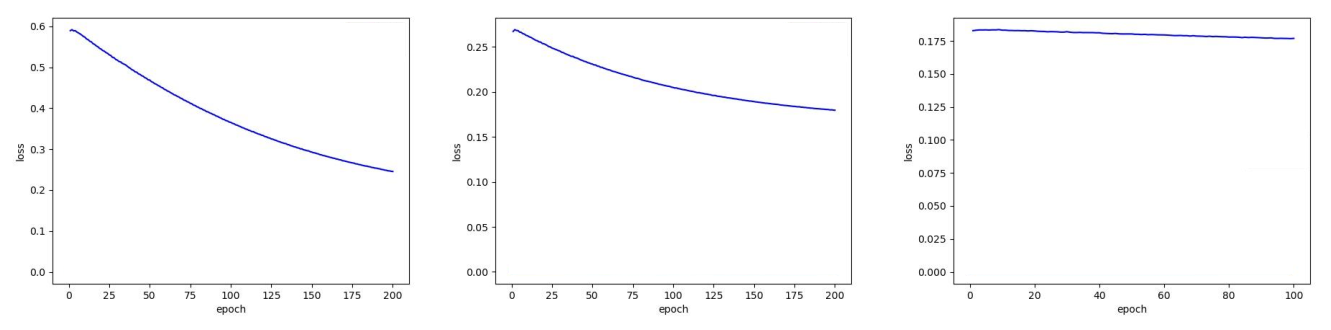
*

**Figure S6**: Training history (loss) of TPGen pre-training. The model is continuously trained in three batches, so there will be three loss plots.

*
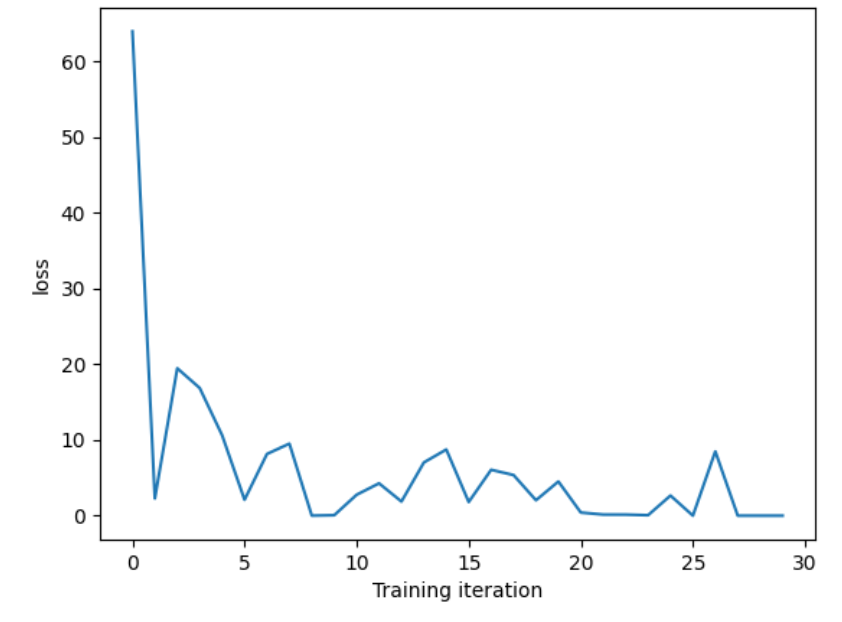
*

**Figure S7**: The training history (loss) of TPGen fine-tuning on the HHH dataset.

*
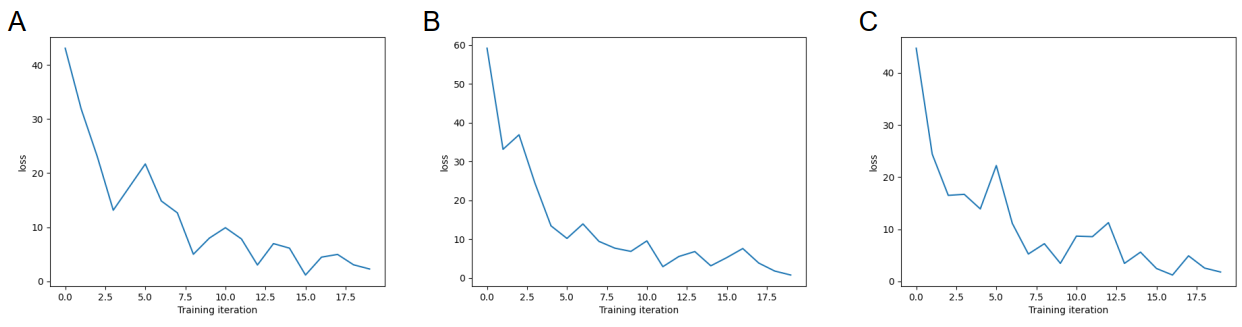
*

**Figure S8**: Train training history (loss) of different fine-tuning strategies. (A) Fine-tuning with λ1=1.0 and λ2=0.0 .(B) Fine-tuning achieved by directly multiplying the stability scores with the discriminative model scores.(C) Fine-tuning with only the discriminative model

*
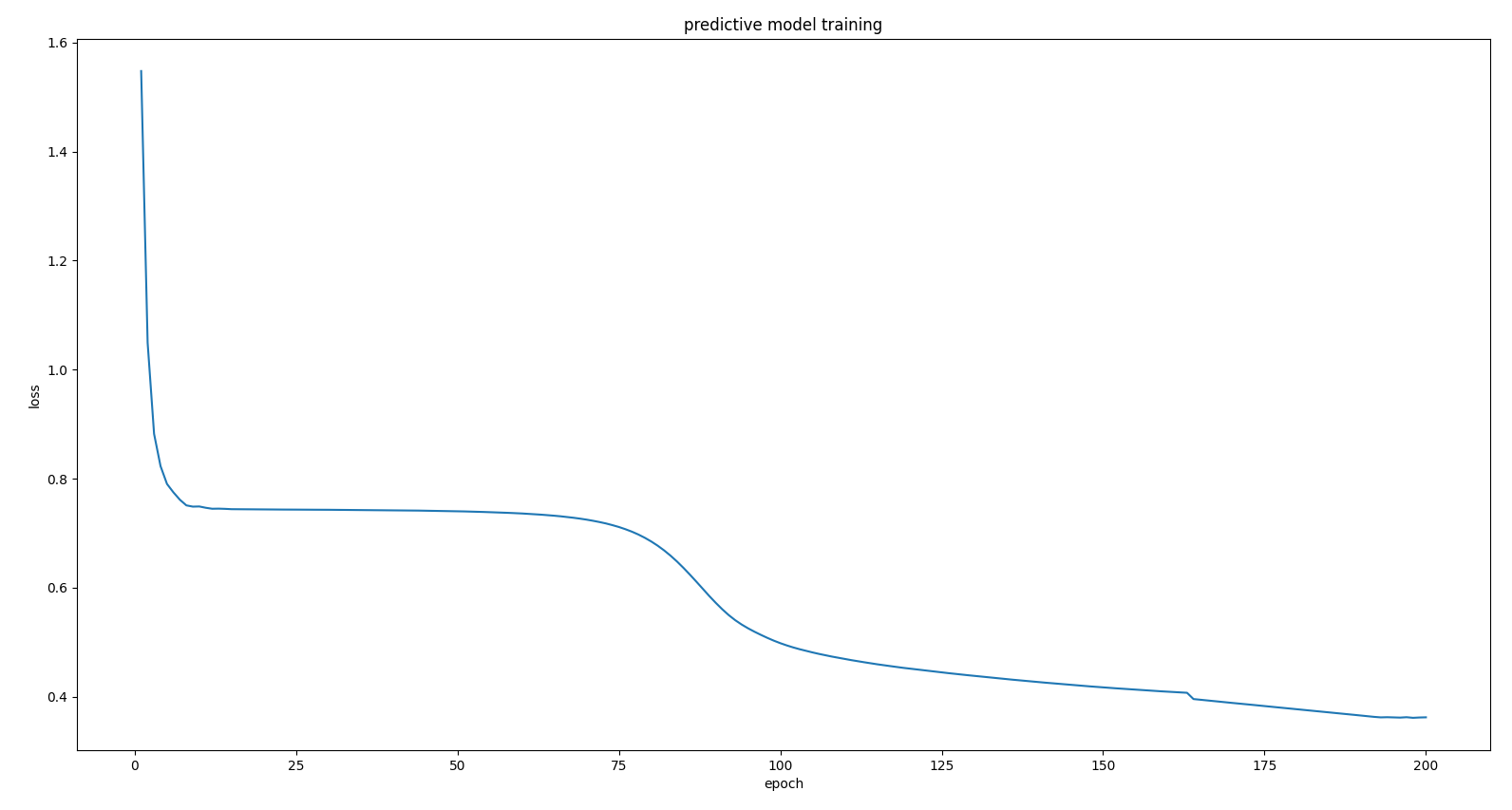
*

**Figure S9**: The training history (loss) of the stability predictive model.


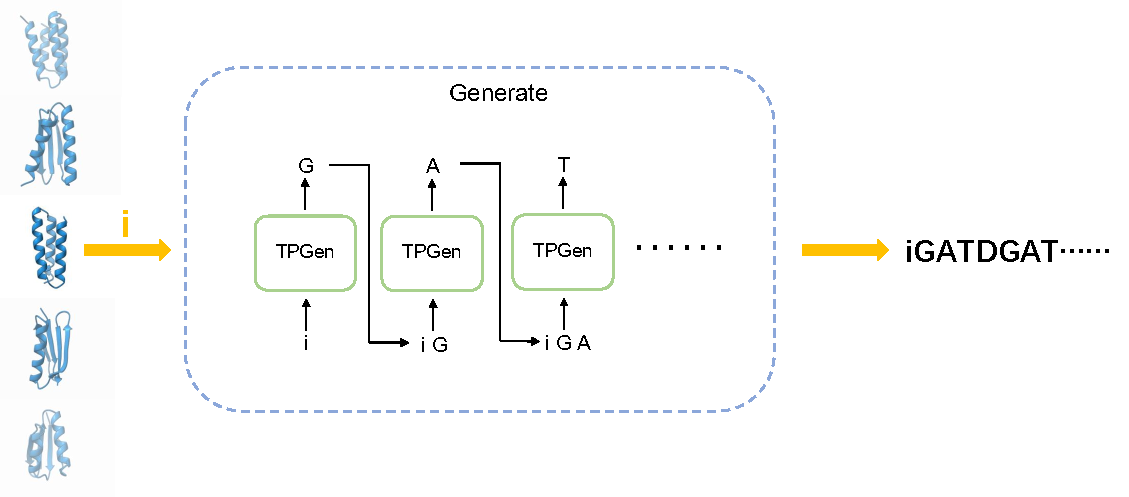


***Figure S10****: A succinct overview of the sequence generation process utilized by TPGen. Upon inputting a designated topological architecture tag, such as 'i' denoting HHH, TPGen embarks on an autoregressive generation sequence that culminates with the emergence of a termination token or upon reaching a predetermined length.*
